# Viral Disruption of Blood–Testis Barrier Precedes Testicular Infection

**DOI:** 10.3390/v17060747

**Published:** 2025-05-23

**Authors:** E. Eldridge Hager-Soto, Alexander N. Freiberg, Shannan L. Rossi

**Affiliations:** Department of Pathology, University of Texas Medical Branch, Galveston, TX 77550, USA; anfreibe@utmb.edu (A.N.F.); slrossi@utmb.edu (S.L.R.)

**Keywords:** testes, blood–testis barrier, pathology, male reproductive health, HIV, mumps virus, Zika virus, Ebola virus

## Abstract

Several viruses have demonstrated the potential for infecting the human male genital tract, leading to potential host pathologic consequences and sexual transmission. Despite the testes being an immune-privileged niche of the body, viruses like Zika, mumps, Ebola, Marburg, and human immunodeficiency virus infect the lumen of testes. The human blood–testis barrier (BTB) is a specialized epithelial barrier responsible for protecting the developing sperm in the lumen of the seminiferous tubules from foreign antigen; however, testicular-tropic viruses possess the unique ability to modulate this barrier prior to entry into the lumen. Previous scientific reports identified immunomodulatory and viral-induced changes to BTB physiology during infection—a necessary step prior to viral entry into the testicular lumen. This review aims to explore the specific mechanisms employed by viruses to disrupt the human BTB and establish testicular infection.

## 1. Introduction

The human testes are a critically protected and immune-privileged organ, responsible for fostering the development and maturation of sperm while producing the androgen testosterone [1,2]. Testes compartmentalize sperm inside seminiferous tubules (STs), a highly organized structure dividing the testicular environment into an immune-privileged lumen and an interstitial space exposed to vasculature and immune cells [3]. The testicular microenvironment is heavily immune-modulated and protected by the blood–testis barrier (BTB), a specialized epithelial structure serving as a physical barrier surrounding the periphery of STs [4]. Positive and negative trade-offs result from these properties of the testes, namely that the sperm are protected from pathogens and autoantigen attack, but pathogens capable of entering the immune-privileged space are therefore shielded from the immune system [5]. To date, several diverse pathogens have demonstrated the ability to exploit this niche, resulting in varying degrees of testicular pathology and potentially risking person-to-person sexual transmission [6,7]. This review will overview the biology of the blood–testis barrier (BTB) and current evidence demonstrating how viruses mechanistically disrupt this barrier, both directly and indirectly, to cause testicular infection.

Strong evidence for viral tropism for testes has emerged since the mid-twentieth century, detailing diverse disease outcomes from poor sperm motility to painful orchitis (inflammation of the testes) [8,9]. Varying degrees of evidence support the potential for many other human viruses to result in testicular infection, underlying the need for further study in this field. Mumps virus (MuV) (*Mumps orthorubulavirus*) was identified as the etiologic agent for epididymo-orchitis in men and continues to be a major concern for infertility amid MMR vaccine hesitancy [9,10]. In the wake of the AIDS pandemic of the 1980s, molecular evidence identified the male genital tract as a critical viral reservoir for human immunodeficiency virus (HIV, *Lentivirus humimdef1*) [11,12,13,14]. Sexual transmission of Marburg virus (MARV, *Orthomarburgvirus marburgense*) was reported during the first recorded human outbreak in 1967 [15,16,17], and epidemics of Ebola, another filovirus (EBOV, *Orthoebolavirus,* multiple species), have provided evidence of sexual transmission as an epidemiologically important transmission pathway [16,18,19,20]. Similarly, Zika virus (ZIKV, *Orthoflavivirus zikaense*) outbreaks in the previous decade have provided supportive evidence of viral sexual transmission, demonstrating that viral testicular infection poses a unique public health concern [18,21,22,23,24,25,26,27]. These findings have spurred an increase in research into viral tropism for testes, leading to key findings for how viruses of diverse families can result in testicular infection and alternative transmission pathways (Table 1). While many gaps in knowledge still pervade this field, emerging evidence points to several overlapping pathways that viruses employ to achieve testicular infection.

Viral invasion of the ST is contingent on two main pathologic events: the delivery of the virus to the testicular space and the subsequent dysregulation of the BTB. The former event can be achieved through either the robust infection of endothelial cells or delivery through a Trojan horse mechanism such as via infected monocytes and/or macrophages [27,28]. The dysregulation of the BTB follows a variety of both viral- and immune-mediated pathways, which will be explored in detail in this review.

**Table 1:** Clinically relevant human viruses discussed in this review with associated cytokines and BTB tight junction proteins.

Virus	Family	Cytokines Associated	Tight Junction Proteins Affected
Mumps virus	*Paramyxoviridae*	TNF-α [29] IL-6 [30] CXCL10 [31]	ZO-1 [29] Occludin [29]
HIV	*Retroviridae*	none *	Occludin [32] Claudin-1 [32] N-cadherin [32] β-catenin [32]
Zika virus	*Flaviviridae*	TNF-α [33] IFN-γ [28]	Claudin-1 [28] ZO-1 [34]
Ebola/Marburg virus	*Filoviridae*	unclear	ZO-1 [35] ZO-2 [35]

* HIV is believed to dysregulate BTB via the viral Tat protein.

## 2. Blood–Testis Barrier

### 2.1. Structure and Function

**Figure 1:**
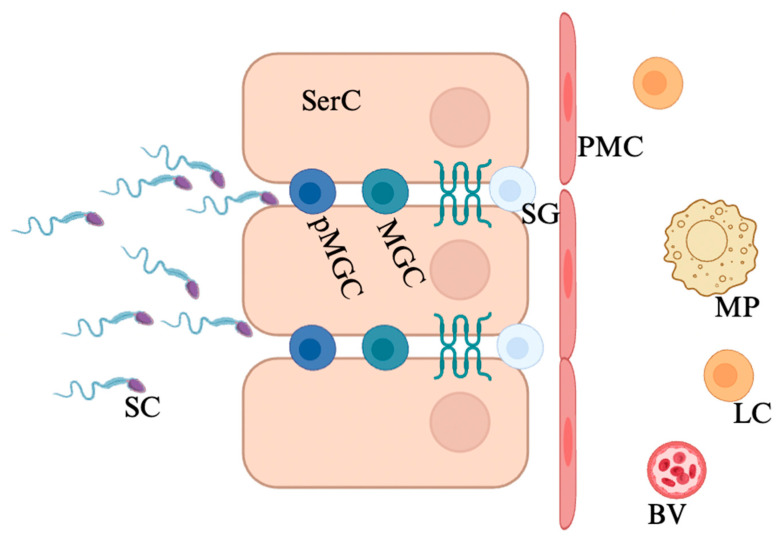
Healthy blood–testis barrier junction organization. The human blood–testis barrier exists in between adjacent Sertoli cells, separating the testicular space into lumen and interstitium. The lumen of the testes is an immune-privileged niche and contains developing sperm cells. SerC: Sertoli cell; PMC: peritubular myoid cell; MP: macrophage; LC: Leydig cell; BV: blood vessel; SG: spermatogonia; MGC: meiotic germ cell; pMGC: post-meiotic germ cell; SC: sperm cell. Figure made with Biorender.

The overall role of the BTB is to shield spermatogenesis from foreign pathogens and autoimmune reactions. The production of sperm occurs only after puberty and the development of self-antigen tolerance by the immune system, making these germ cells targets for the immune system, which views sperm as foreign antigen [36]. As a result, the introduction of sperm into any non-testicular tissue results in an autoimmune reaction [37,38]. Therefore, the BTB serves as a critical barrier to prevent the escape of sperm from the testicular niche or any immune activity inside the STs. Human testes are organized into two discrete regions—the lumen of STs and interstitium—separated by the BTB, which is formed between adjacent Sertoli cells (Figure 1). Sertoli cells are a testicular-specific epithelial cell responsible for maintaining the integrity of the BTB in addition to nursing developing germ cells to full maturity [39,40]. The lumen of the STs contains all developing germ cells, and the interstitial space between STs contains all vasculature, immune cells such as resident testicular macrophages and dendritic cells, and testosterone-producing Leydig cells (Figure 1) [41]. The BTB separates these distinct tissue regions, keeping the lumen of the STs immune-privileged. Evidence for the existence of the BTB first emerged in the mid-twentieth century, when researchers noted differences in dye penetration in the testes of prepubescent and pubescent rats [42,43].

The BTB is formed through the expression of a highly regulated arrangement of four unique categories of cellular junctions in between Sertoli cells: tight junctions, gap junctions, desmosome-like junctions, and ectoplasmic specializations [39,44,45,46]. This complex assortment of diverse tissue junctions sets the BTB apart from other tissue barriers like the blood–brain barrier (BBB) and makes the BTB one of the strongest tissue barriers [47]. In addition to serving as an immunologic divider for STs, the cell polarity in a mature BTB creates an apical and basal side of the epithelium. This cellular polarity is critical for the completion of meiosis, since the migration of developing sperm in a basal-to-apical direction is essential for the delivery of mature sperm into the seminiferous tubule lumen [48,49]. Spermatogonia and preleptotene spermatocytes exist on the basal side of the BTB and are exposed to the lymphatic and circulatory system until these cells mature further and migrate toward the seminiferous tubule lumen in later stages of meiosis. Sertoli cells modulate their own barrier integrity to allow this cellular migration with the downside that viruses can exploit these same mechanisms to allow BTB weakening and entry [28,29,35]. Despite this regular restructuring of the BTB, the protective function of the barrier is maintained and protects the lumen of the seminiferous tubules from viral infection during healthy spermatogenesis [50]. This process of cellular transport during meiosis is still not fully understood, and consequently, many gaps remain in elucidating how viruses mechanistically dysregulate BTB integrity.

### 2.2. Cellular Junctions of the Blood–Testis Barrier

**Figure 2:**
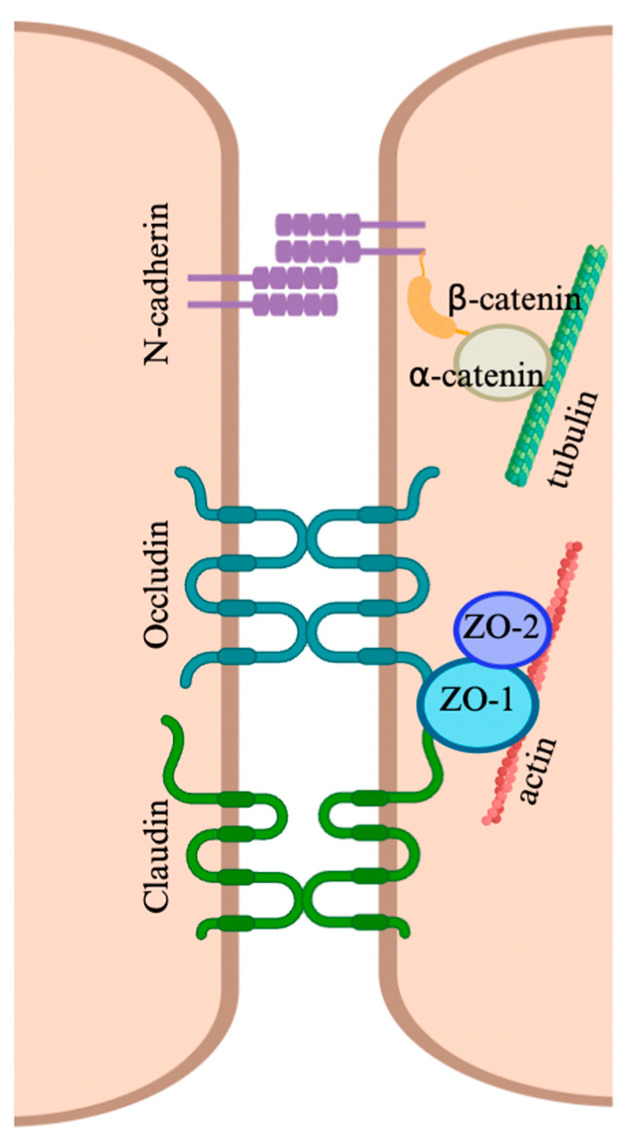
Organization of key tight junction proteins found at the blood–testis barrier. Claudins, occludin, and N-cadherin contain extracellular domains that interact with neighboring cells. Intracellularly, these integral membrane proteins interact with scaffolding proteins that are expressed entirely intracellularly (ZO-1, ZO-2, α-catenin, and β-catenin). Scaffolding proteins are responsible for linking the embedded integral membrane tight junction proteins to the cellular cytoskeleton. ZO-1: zonula occludens-1; ZO-2: zonula occludens-2. Figure made with Biorender.

Tight junctions, gap junctions, desmosome-like junctions, and ectoplasmic specializations each comprise unique junction proteins. Tight junctions are mainly composed of membrane proteins, like claudins, occludin, and tricellulin, and intracellular scaffolding zonula occludens proteins—though the BTB is not limited to expressing these tight junction proteins, with others playing a more minor role [51,52,53,54,55,56,57,58,59,60,61,62]. Claudins include 24 different transmembrane proteins organized under the claudin family umbrella, marked by their ability to form junctions with other claudin proteins. Interestingly, some claudins can additionally serve as receptors or binding factors for various pathogens such as HIV and *Clostridium perfringens* toxin (Figure 2) [63,64]. Occludin is similar to claudins in that it is a transmembrane protein that forms junctions with neighboring cells, but unlike claudins, it does not belong to a large family of structurally similar proteins. Occludin only associates with other occludin proteins on adjacent cells and is considered one of the major contributors to BTB strength in addition to regulating Sertoli cell polarity (Figure 2) [65,66]. Tricellulin is a similar tight junction protein, though specializing in tricellular junctions, as its name suggests [67]. As opposed to these three structural proteins, the zonula occludens (ZO) protein family is expressed intracellularly and acts as scaffolding proteins. These proteins mainly serve to anchor transmembrane proteins such as occludin and claudin family proteins to the actin cytoskeleton of the cell (Figure 2). These proteins exist in many domains, but mainly ZO-1 and -2 are associated with the BTB [60,61]. Gap junctions, desmosome-like junctions, and testis-specific ectoplasmic specializations are all essential to the function of the BTB; however, they have not yet been associated with viral dysregulation of the BTB and will therefore not be explored in detail in this review.

### 2.3. Permeability Regulation

Various endocrine and paracrine factors act to strengthen or weaken the permeability of the BTB for the ingress of developing sperm cells, and viral pathogens can exploit these factors to enter the immune-privileged ST lumen. Endocrine factors mainly include hormones responsible for BTB formation during the onset of spermatogenesis at puberty, such as follicle-stimulating hormone (FSH) and luteinizing hormone (LH) [68]. The suppression of gonadotropins causing a decrease in FSH and LH is associated with a loss of tight junction strength at the BTB in postpubescent adults, indicating that the maintenance of these hormones is necessary [69]. Though these endocrine regulators directly affect BTB permeability, more local paracrine factors help regulate the restructuring of the BTB for germ cell migration. These factors are expressed by several testicular cells including Sertoli cells as well as testicular interstitial cells like resident testicular macrophages and Leydig cells, which are responsible for local immune modulation and producing testosterone, respectively. The transforming growth factor-β (TGF-β) superfamily has been shown to weaken the BTB, most likely through inhibiting the expression of various tight junction proteins such as occludin, members of the claudin family, and ZO-1 [70]. This cytokine most likely operates through the induction of the p38 MAP kinase pathway, which can thereby affect cellular tight junction protein expression; however, the overall mechanism remains largely unclear [70]. By contrast, retinoic acid expression in the BTB is associated with the tightening of the barrier and is observed at Sertoli cell junctions during germ cell translocation [71]. Many of these cytokines, like TGF-β, are typically constitutively expressed by Sertoli cells at different levels to allow the progressive weakening and strengthening of specific regions of the BTB for germ cell migration during healthy meiosis. The expression of testosterone in the testicular interstitium is also associated with BTB permeability. The binding of testosterone to its receptor on Sertoli cells is associated with a decrease in claudin-3 expression, contributing to a decrease in BTB permeability [59,72]. Lastly, various pro-inflammatory cytokines expressed by both Sertoli cells under stress and immune cells residing in the testicular interstitium can impact the permeability of the BTB. Tumor necrosis factor-α (TNF-α) expressed during inflammation can decrease BTB permeability through a similar mechanism to TGF-β via activation of protein kinases [73]. Interleukin (IL)-6 and -17 have also been strongly associated with decreasing BTB integrity. The expression of these cytokines is related to the dysregulation of BTB integrity, and the introduction of both IL-6 and IL-17 caused a decrease in BTB strength and the redistribution of claudins and occludin [74,75,76]. Questions remain in understanding which of these local effectors of BTB permeability are intentionally expressed for barrier restructuring during germ cell translocation and which cause incidental changes in permeability.

## 3. Viral Infection of Testes

### 3.1. Mumps Virus

Mumps virus (MuV, *Paramyxoviridae*) is the etiological agent of mumps, a vaccine-preventable disease typically associated with painful inflammation of the salivary glands, headache, and fever. MuV is also neurotropic, causing infection of the central nervous system in nearly half of tested patients [77]. Notably, orchitis is the second-most common symptom in postpubescent males, after swelling of the salivary glands [78,79]. Testicular atrophy occurs to some extent in approximately half of cases where orchitis occurs, and this is associated with fertility complications like oligospermia; however, full sterility is an exceptionally rare outcome [80,81]. Mumps typically self-resolves within a week to a month, but infection can result, albeit rarely, in long-term neurologic sequelae involving seizures and deafness. Though mumps is vaccine-preventable, MuV is considered a re-emerging pathogen due to recent outbreaks related to vaccine hesitancy [82].

The dynamics of MuV infection in testes have been greatly studied due to the high incidence of orchitis in infected postpubescent males. MuV remains detectable in semen for long periods of time post-infection—up to over a month after the onset of symptoms—and infectious MuV has been isolated from the testicular tissue of patients via fine-needle aspiration biopsy, heavily suggesting the direct infection of testicular cells [10,83]. Sertoli cells have demonstrated susceptibility to MuV infection both in vitro and in vivo in a mouse animal model, suggesting that Sertoli cells are a potential target for MuV testicular infection [29]. MuV-induced orchitis is associated with reduced testosterone production by Leydig cells; however, this likely does not contribute to MuV invasion of the STs since lower testosterone expression is not associated with a weakened BTB [84,85]. Due to the self-limiting, non-lethal nature of MuV infection, obtaining intact pathological samples is difficult, limiting our understanding of MuV testicular invasion.

**Figure 3:**
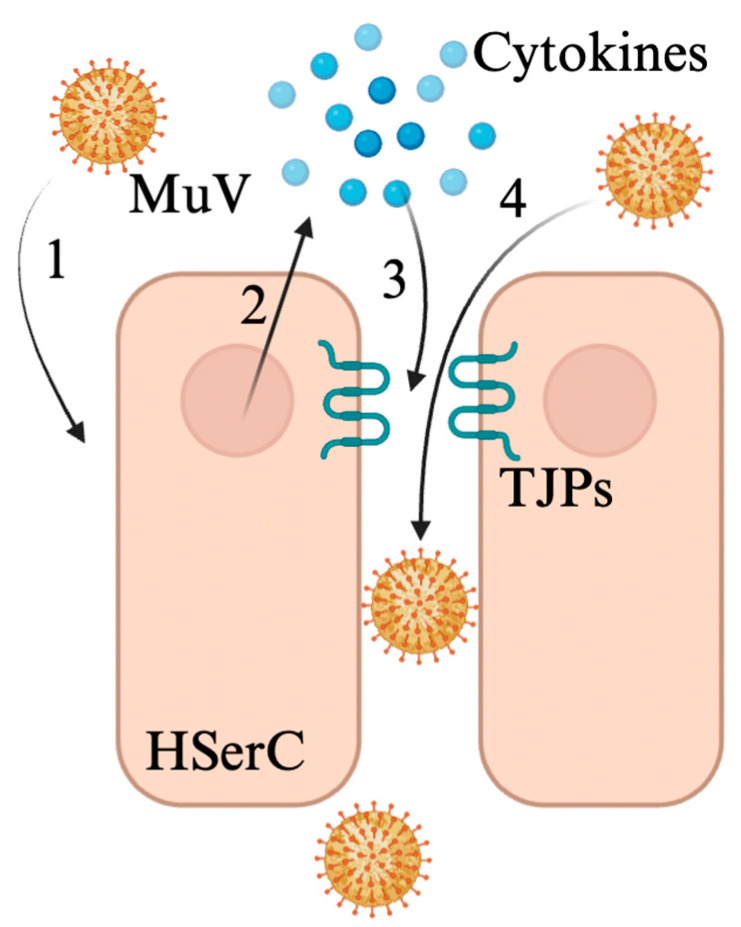
Mumps virus invades the testicular lumen through 4 main events. 1. Infection of Sertoli cells by MuV in the interstitium. 2. Expression of pro-inflammatory cytokines by Sertoli cells including TNF-α, IL-6, and CXCL10. 3. Disruption of tight junction proteins (TJPs) zonula occludens-1 and occludin by these cytokines. 4. Introduction of MuV virions through a leaky BTB. Figure made with Biorender.

Despite the lack of clinical samples, in vitro and in vivo data illustrate potential mechanisms surrounding MuV entry into Sertoli cells and the subsequent dysregulation of the BTB (Figure 3). Sialic acid has been shown to mediate MuV entry into Sertoli cells via binding to receptor tyrosine kinases AXL and MER, further serving as potential binding sites for MuV on Sertoli cells [30]. The Sertoli cell response to MuV infection includes the expression of several pro-inflammatory cytokines that may contribute to BTB disruption during infection, such as TNF-α, IL-6, and CXCL10 [29,30,31]. Exposure of the BTB to TNF-α is classically associated with decreased BTB permeability, and MuV infection follows this pattern. The immunostaining of occludin and ZO-1 in healthy and MuV-infected mice demonstrates a clear disruption of two key tight junction proteins of the BTB during active viral infection [29]. Taken together, these data suggest that MuV impairs BTB permeability mainly through an immune-mediated pathway, with pro-inflammatory cytokines serving as the main effectors.

### 3.2. Human Immunodeficiency Virus

Human immunodeficiency viruses (HIV) include two human lentivirus species (*Lentivirus humimdef 1* and *Lentivirus humimdef 2*) that are enveloped, single-stranded, positive-sense RNA retroviruses [86]. Disease caused by HIV initially presents as fever and other flu-like symptoms, accompanied by swelling of lymph nodes and tonsils; however, late-stage disease results in acquired immunodeficiency syndrome (AIDS), leaving patients vulnerable to otherwise non-threatening infections due to a weakened immune system. Patients survive an average of ten years after time of infection with HIV [87]. The viral load of HIV in patients gradually increases after each year, significantly increasing after the development of AIDS, resulting in a higher rate of transmission [88]. There is no approved vaccine preventing HIV infection; however, antiretroviral therapy is successful at reducing viral replication throughout a patient’s life, making HIV a manageable infection, rather than uniformly lethal for patients receiving care [89].

HIV testicular infection has been extensively studied due to its high morbidity and mortality, spreading through sexual transmission. HIV infection is associated with decreased levels of spermatogenesis and testicular atrophy [14]. Testicular tissue shows evidence of HIV infection in the autopsy samples and semen of patients with HIV at high prevalence. In addition to hypogonadism, the pathological consequences of HIV infection include the shrinking of the seminiferous tubule diameter, the thickening of the testicular basement membrane, fibrosis observed in the interstitial space, perivasculitis, and an increased vulnerability to opportunistic infections [90,91]. Testes have also been identified as a likely site for prolonged HIV infection [92]. Unique among most testicular-tropic viruses, HIV does not infect testicular-specific cells; rather, HIV infects CD4+ lymphocytes, macrophages, and dendritic cells that reside in low numbers in the testicular microenvironment [13,14,93]. The scientific literature reports conflicting results regarding whether HIV can infect germ cells. HIV has been detected in spermatogonia and sperm cells, but the extent to which the virus can productively replicate in these cell types has been called into question, mostly since these cells lack the putative receptor for HIV [14,94,95].

**Figure 4:**
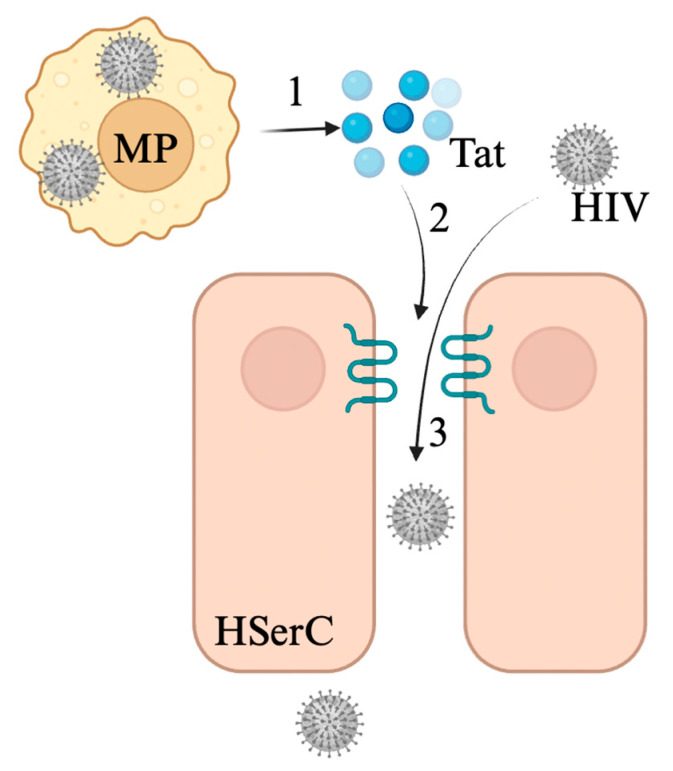
Overview of HIV invasion of seminiferous tubule lumen. 1. HIV-infected macrophages, monocytes, CD4+ T cells, and/or dendritic cells express tat protein. 2. Tat protein disrupts BTB through dysregulation of claudin-1, occludin, N-cadherin, β-catenin, and vimentin. 3. HIV can pass the BTB. Figure made with Biorender.

HIV-infected cells secrete transactivating regulatory protein (Tat) which contains a cell-penetration peptide that likely contributes to the weakening of the BBB, hinting at similarities in mechanism of action between the BBB and BTB [32,96,97]. In vitro studies found a strong association between the dysregulation of the Sertoli cell microtubule cytoskeleton and the dysregulation of BTB tight junction proteins such as N-cadherin, occludin, zonula occludens-1, and β-catenin with the presence of Tat protein (Figure 4) [32]. Furthermore, immune cells permissive to HIV infection are implicated in secreting cytokines such as TGF-β that are associated with BTB permissiveness [32,98]. The secretion of these cytokines during active HIV infection likely contributes to the leakiness of the BTB seen in patients with HIV. These findings accompanied with the high incidence of HIV positivity in testes suggest that the testicular environment remains a sanctuary for HIV infection, causing lasting leakiness in the BTB and the infection of HIV virions in sperm [13].

### 3.3. Zika Virus

Zika virus (ZIKV) is an orthoflavivirus primarily spread by *Aedes*-species mosquitoes across the global tropics. ZIKV rose to prominence in the wake of the 2015–2016 epidemic resulting in an estimated more than 1,000,000 cases of ZIKV in the Americas, mostly Brazil, a previously non-endemic region [99,100,101]. Infections are typically asymptomatic, but the clinical presentation of ZIKV disease includes fever, maculopapular rash, headache, arthralgia, myalgia, and nonpurulent conjunctivitis [102,103]. ZIKV is an infection that typically self-resolves within 1–2 weeks after the onset of symptoms. Notably, infection can result in Guillain–Barré syndrome and neurological complications as a rare outcome in adults. Congenital ZIKV syndrome, a fetal developmental abnormality resulting in malformations such as microcephaly, eye abnormalities, and deafness, is another matter of high concern [104,105].

The first published evidence for ZIKV sexual transmission was reported in 2008, describing a case of male-to-female viral transmission in Colorado, USA, in the absence of the *Aedes* vector [106]. After the increase in case number following the 2015–2016 epidemic, mounting epidemiological and molecular evidence pointed to the sexual transmission of ZIKV as a possible epidemiological pathway. ZIKV is capable of establishing long-term infection of testes, with the infectious virus or viral genome detectable via PCR isolated from semen up to 69 days and 304 days after the onset of symptoms, respectively [107,108]. Biopsy samples of ZIKV-infected testes are rare since most adult patients recover, requiring the use of animal models to determine the cell types affected and the mechanisms of infection. Murine models of ZIKV infection show testicular damage, including the shrinking of seminiferous tubules and testicular atrophy; however, to what extent this applies to human patients is not known [109,110,111]. Clinically, genitourinary symptoms have been reported in patients including painful ejaculation, prostatitis, and oligospermia [112].

**Figure 5:**
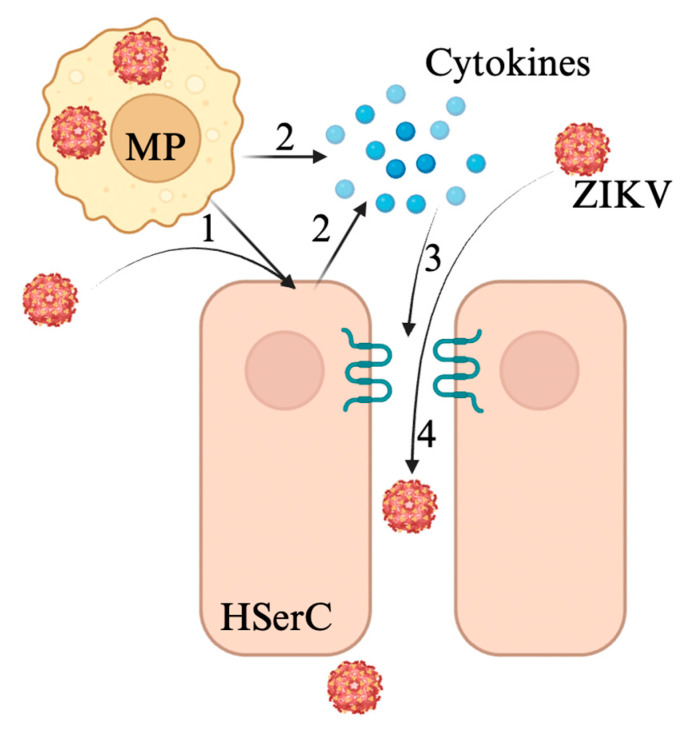
ZIKV disrupts the BTB to cross into the lumen in 4 main steps. 1. Infection of Sertoli cells by ZIKV either through viral release by infected macrophages or through blood. 2. Release of TNF-α and IFN-γ by infected Sertoli cells and/or infected macrophages. 3. Disruption of claudin-1 by these pro-inflammatory cytokines. 4. ZIKV can then cross into the lumen, potentially also accompanied by ZIKV-infected macrophages. Figure made with Biorender.

ZIKV testicular infection is well studied, with numerous studies demonstrating ZIKV infection of Sertoli cells binding to the AXL receptor and invasion of testicular lumen in murine models [109,113,114,115]. Mechanistically, ZIKV infection in the testicular microenvironment has received less attention, but some studies offer insights into how ZIKV crosses the BTB (Figure 5) [28,115,116]. ZIKV envelope protein has been associated with the rearrangement of the actin cytoskeleton of Sertoli cells, affecting BTB integrity in the context of ZIKV infection [34]. An analysis of Sertoli cell physiology during ZIKV infection showed the dysregulation of ZO-1 as well as exposure to isolated envelope protein, suggesting direct modulation by the ZIKV envelope protein [34]. Further evidence suggests that ZIKV infection of Sertoli cells is a critical step due to the subsequent induction of TNF-α, which thereby induces a weakening of BTB permeability [33]. Furthermore, ZIKV-infected S100A4+ macrophages, a myeloid macrophage subpopulation highly susceptible to ZIKV, have been strongly implicated in this process, as the secretion of IFN-γ was correlated with the translocation of the tight junction protein claudin-1 in the context of ZIKV infection of a mouse model [28]. ZIKV may therefore demonstrate both virus- and immune-mediated pathways toward the penetration of the BTB during testicular infection.

### 3.4. Ebola and Marburg Viruses

The Ebola (EBOV) and Marburg viruses (MARV) are two hemorrhagic RNA viruses from the Filoviridae family that both cause severe disease in humans. EBOV and MARV were first recognized in 1976 and 1967, respectively, and have since sporadically resulted in human outbreaks, mainly localized to sub-Saharan, Central, and West Africa. Outbreaks of these viruses carry high mortality rates, ranging from 24 to 90%, due to differences in outbreak size and public health response [27,117,118]. EBOV and MARV display similar pathways of entry, dissemination, and pathology. EBOV/MARV enter the body through mucous membranes or skin lesions and target monocyte lineage cell types like dendritic cells, monocytes, and macrophages [119,120,121,122,123,124]. After achieving high-level replication in these cell types, the virus disseminates throughout the body, infecting many tissue and organ types, causing immune overreaction and coagulopathies [125,126]. Initial symptoms include lethargy, nausea, abdominal pain, and vomiting. In severe cases, organ failure and death result after approximately ten days, but the administration of supportive care improves disease outcomes [123,127]. EBOV Zaire is vaccine-preventable, and additional vaccines are being developed and approved against more strains of EBOV and filoviruses [128,129,130].

The sexual transmission of MARV has been considered since its onset in 1967, with one female patient in the initial 31-patient outbreak becoming infected through sexual intercourse with her husband two months after his hospital release [15,131,132]. Evidence for the sexual transmission of EBOV first emerged in the aftermath of the 1995 outbreak in Kikwit, Democratic Republic of the Congo, resulting in 315 cases and 71 survivors [133,134]. Several male survivors were monitored after the resolution of disease, with EBOV remaining detectable via PCR in semen for months after symptom onset [133,134]. There is a paucity of in-depth pathological analysis of EBOV and MARV in human testes, but infected testes have typically displayed endothelial damage, fibrin clots, and thrombocytopenia [135]. Analysis of non-human primate models for EBOV and MARV finds extensive testicular infection and varying levels of inflammation in the testes [136,137]. Examples of EBOV transmission months or years after the resolution of symptoms underline the significance of this tissue tropism for testes in propagating further epidemics [19,20]. The literature lacks direct evidence to describe EBOV or MARV entry into Sertoli cells; however, Sertoli cells do express AXL, a member of the Tyro3 receptor tyrosine kinase family, which can be used by both EBOV and MARV for cellular attachment and entry [138].

**Figure 6:**
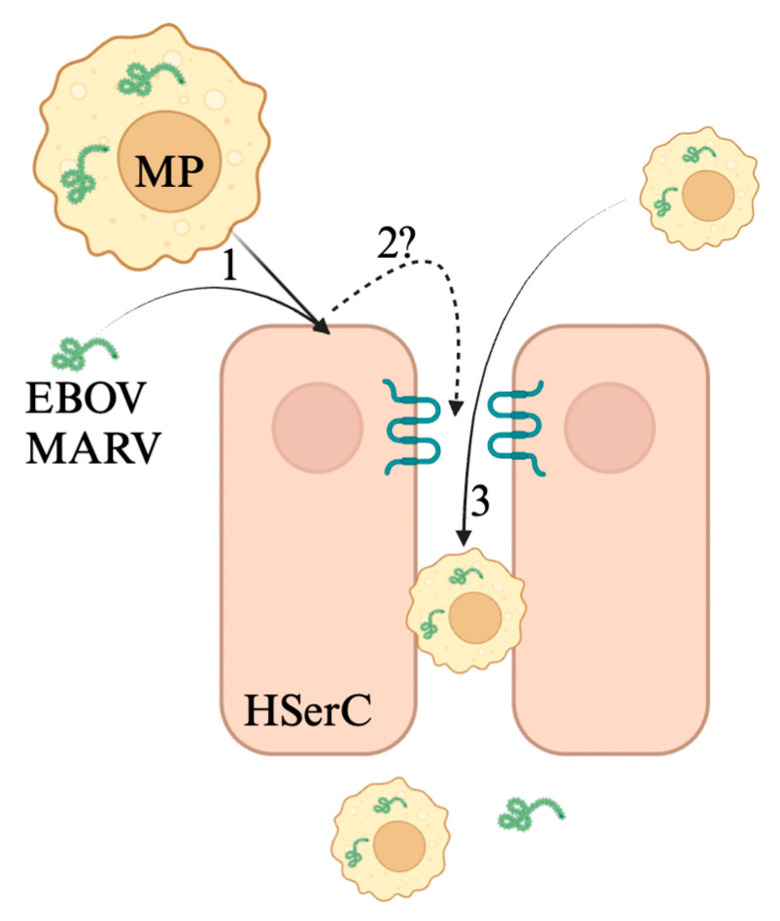
MARV and EBOV invade the testicular lumen through a less-understood mechanism. 1. Sertoli cells are susceptible to both MARV and EBOV, delivered by both circulating virus in blood and infected macrophages. Zonula occludens-1 and zonula occludens-2 are disrupted during MARV infection. The mechanism behind this is unclear. This leaves the BTB leaky, allowing infected macrophages (3) to pass into the lumen of the seminiferous tubule. It remains unclear whether virions are capable of crossing the weakened BTB during filovirus infection of the testes (2?). Figure made with Biorender.

Non-human primate research in EBOV and MARV pathology offers several insights into potential mechanisms by which these filoviruses invade the testicular microenvironment. A 2018 study on rhesus monkeys infected with MARV found that testicular infection with MARV was accompanied with high recruitment of macrophages that infiltrated the BTB [35]. This study also found a high degree of Sertoli cell infection and degradation of ZO-1 and ZO-2 in seminiferous tubules [35]. These data suggest that MARV infection in the testicular microenvironment triggers macrophage recruitment, Sertoli cell infection, and diminished expression of BTB tight junction proteins (Figure 6). Research on EBOV infection of the testes shows infection in the testicular microenvironment, past the BTB, in the lumen of the testes in humans and non-human primates [135,136]. *In vitro* data demonstrate the susceptibility of Sertoli cells to EBOV, and murine data indicate EBOV detection in the periphery seminiferous tubules [139,140]. Together, these findings demonstrate similar parallels to Marburg virus infection, but further research is required to draw more insights into potential mechanisms behind EBOV infection of the testes.

## 4. Conclusions

Viral infection of the testicular microenvironment shares several common mechanisms of interest. For all viruses discussed in this review, some mechanism of weakening the BTB is required prior to viral entry into the testicular lumen. The expression of pro-inflammatory cytokines triggering the weakening of the BTB occurs during both MuV and ZIKV infection. The involvement of macrophages either through viral delivery to the testicular space, the expression of cytokines, or both is common among HIV, ZIKV, MARV, and EBOV infection. HIV and potentially ZIKV have shown viral-induced mechanisms of BTB disruption rather than solely immune-mediated disruption.

More viruses are poised to join this list, with several emerging infectious diseases showing signs of testicular infection. In the wake of the COVID-19 pandemic, researchers have demonstrated SARS-CoV-2 infection of testes in animal models and have mounted strong clinical evidence that testicular pathology can result from COVID-19 disease in humans [141,142]. A patient who succumbed to infection by Powassan virus (*Orthoflavivirus powassanense*), an emerging tick-borne virus, reported testicular pain, and viral antigen was detected in the lumen of the seminiferous tubules [143]. Additionally, Oropouche virus (*Orthobunyavirus oropoucheense*), an emerging arthropod-borne virus spread by biting midges, was recently identified in the semen of a patient sixteen days after the onset of symptoms [144]. Given the parallels identified in the testicular-tropic viruses discussed in this review, these viruses may cross the BTB using similar means. This diverse array of medically relevant viruses recently implicated in being testicular-tropic raises questions of the potential for viral testicular infection being a more common feature than previously assumed, necessitating further research.

Interestingly, most of the testicular-tropic viruses also display a tropism for other immune-privileged niches of the body, including the eye and brain. For example, EBOV is associated with causing neurological sequelae and ocular pathology, in some cases lasting long after the resolution of acute disease [25,145]. ZIKV can also cause ocular pathology, and MuV can cause neuropathology and has been detected in brain and CNS fluid [25,77]. Despite the physiological differences between the BTB, blood–ocular barrier, and BBB, many pathways of the strengthening and weakening of barrier integrity are conserved among all three. Pro-inflammatory cytokines like TNF-α and IL-6 deleteriously impact the integrity of these three barriers. Furthermore, macrophages have access to these immune-privileged spaces. Together, these findings demonstrate potential parallel mechanisms behind how viruses cross these specialized tissue barriers.

## Data Availability

No new data were created or generated for this study. Data sharing is not applicable to this article.
